# Changes in expectation impact multiple steps of the visual perceptual decision process in adults

**DOI:** 10.14814/phy2.70716

**Published:** 2026-01-29

**Authors:** Julien Audiffren, Jean‐Luc Bloechle, Jean‐Pierre Bresciani

**Affiliations:** ^1^ CopeLAB, University of Fribourg Fribourg Switzerland

**Keywords:** antisaccades, expectation, reaction time, rise‐to‐threshold, saccades

## Abstract

Perceptual decision‐making processes, particularly in the context of eye movements and reaction times (RT), have been studied to better understand how the brain integrates and responds to sensory information. Recent models have decomposed the process into multiple intermediate steps, including detection, instruction processing, decision, and motor response. To investigate the impact of the observer's expectations on each of these steps, we conducted two experiments on 24 participants (including both female and male participants), manipulating respectively the stimuli's location expectation (left or right) and the eye movement expectation (saccade or antisaccade). The results revealed limited evidence for the influence of location expectation on saccadic RT and moderate evidence for antisaccadic RT. Conversely, there was strong evidence of the influence of movement expectations on both movements’ RT. This suggests an asymmetric impact of expectations on the different steps of perceptual decision‐making, with strong impact on motor response and instruction processing. These findings challenge the common attribution of expectation effects solely to the decision‐making module from previous works, emphasizing the importance of considering multi‐module integration in perceptual decision models.

## 1 INTRODUCTION

Delays in perceptual decisions have attracted the interest of the research community for a long time. A commonly studied example is the latency of ocular saccades, or Saccadic Reaction Time (SRT), which usually lies between 200 and 250 ms. Such latency is substantially longer than the theoretically minimal time required for information to propagate to and from the superior colliculus—the shortest possible physiological pathway for a saccade, estimated to be approximately 60 ms (Guitton, 1992). This delay is assumed to be necessary for the brain to interpret the different stimuli and *decide* what to look at. Indeed, without the intervention of higher structures, the superior colliculus could not determine what the different stimuli are, and without such mechanisms, the visual system could be overwhelmed with noise and less relevant stimuli (Carpenter, 1999). In addition to their increased latency, SRT has also been shown to be highly variable, even in experiments where the stimuli have been carefully controlled to be identical (Noorani & Carpenter, 2016). This phenomenon is not trivially explained by the previous considerations, but has been suggested to originate from a need for randomness in decision‐making (Carpenter, 1999).

**FIGURE 1:**
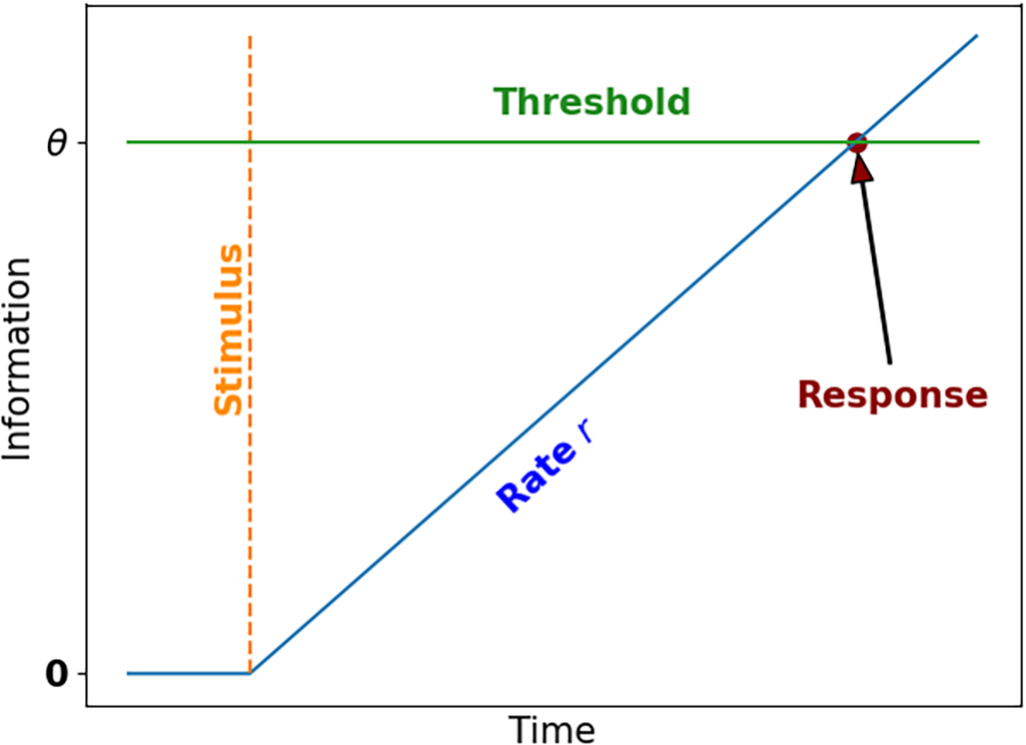
Illustration of the LATER rise‐to‐threshold model. The amount of information starts at zero, and when the stimulus appears it accumulates at the rate r until the threshold θ is reached, at which point a response occurs.

To better understand these neurophysiological mechanisms, multiple competing models have been proposed to approximate the SRT and explain their properties (see e.g., (Brown & Heathcote, 2008; Carpenter & Williams, 1995; Usher & McClelland, 2001; Vickers, 1979) to name only a few). In particular, Rise‐to‐threshold is a popular class of models for perceptual decision‐making (Nakahara et al., 2006). In these models, information regarding the stimuli is perceived and then integrated over time (the rise) until enough has been collected to reach a decision (the threshold). Interestingly, there is some evidence that the information is collected and processed in the brain in the form of log‐likelihood ratio, a common feature across multiple models (Gold & Shadlen, 2001). Despite their apparent simplicity, these models have shown great results in modeling and interpreting a wide range of SRT phenomena (Taylor et al., 2006). In that regard, the LATER model (Linear Approach to Threshold with Ergodic Rate) has been shown to be particularly successful and flexible. This is notably because, despite having only two parameters in its simplest form, it is able to capture many aspects of response latencies (Noorani & Carpenter, 2016). The original LATER model is based on the observation that the inverse of the SRT, called the rate of information acquisition r, appears to follow a normal distribution of mean μ and variance $\sigma^{2}$, that is,

$$r=\frac{1}{\mathrm{SRT}}\propto N\left(\mu ,\sigma^{2}\right),$$

where μ,σ are the two parameters of the model. From a rise‐to‐threshold perspective, the decision process is assumed to start from zero, acquire information at rate r and induce a response when the threshold θ is reached (see Figure 1). Multiple works have investigated the nature of each of the model's parameters (μ, $\sigma^{2},$ and θ). Experiments have shown that manipulating the rate of information supply (e.g., the cohesion of RDK) influences μ (Reddi et al., 2003), or that increasing the task's urgency reduces θ (Reddi & Carpenter, 2000); see (Noorani & Carpenter, 2016) and references therein for a discussion of the impact of each parameter.

In many SRT experiments, the observer is asked to look at a fixation point, until a stimulus appears in one of several possible locations, at which point the observer must perform a saccadic movement toward it. Multiple works have investigated the impact of manipulating the probability of appearance of the stimulus at different locations in this experiment. Indeed, if the target appears disproportionally often on one particular place, it may lead to increased expectation (also called *prior knowledge*, or priors for short) of the observer that may impact SRT. Indeed, in (Carpenter & Williams, 1995) the authors showed a direct link between the log likelihood probability of the stimulus appearing to at a given place and the SRT–and in particular θ (the amount of information necessary to reach a decision). They estimated the impact around −80 ms of reaction time per log unit of prior probability. In line with this result, (Basso & Wurtz, 1997) have shown that the neural activity in the superior colliculus reflects uncertainty in the target apparition, offering a neurophysiological explanation for this phenomenon. Following works (Basso & Wurtz, 1998; Dorris & Munoz, 1998) have also investigated the impact of unbalanced priors in monkeys, although the size of the impact they measured was significantly smaller, for example, ≈266 ms with uniform uncertainty, and ≈245 with the same target constantly chosen in (Basso & Wurtz, 1998). More recently, (Anderson & Carpenter, 2006) studied the impact of priors as well as the gradual change in expectation in human observers, reaching an estimation of ≈−29 ms/log unit. Furthermore, they claimed that 70 trials were enough for the observers to acclimate to a new prior, and that the update of expectations could be modeled with an exponential decay, similar to the Q‐learning algorithm (Watkins & Dayan, 1992).

**FIGURE 2:**
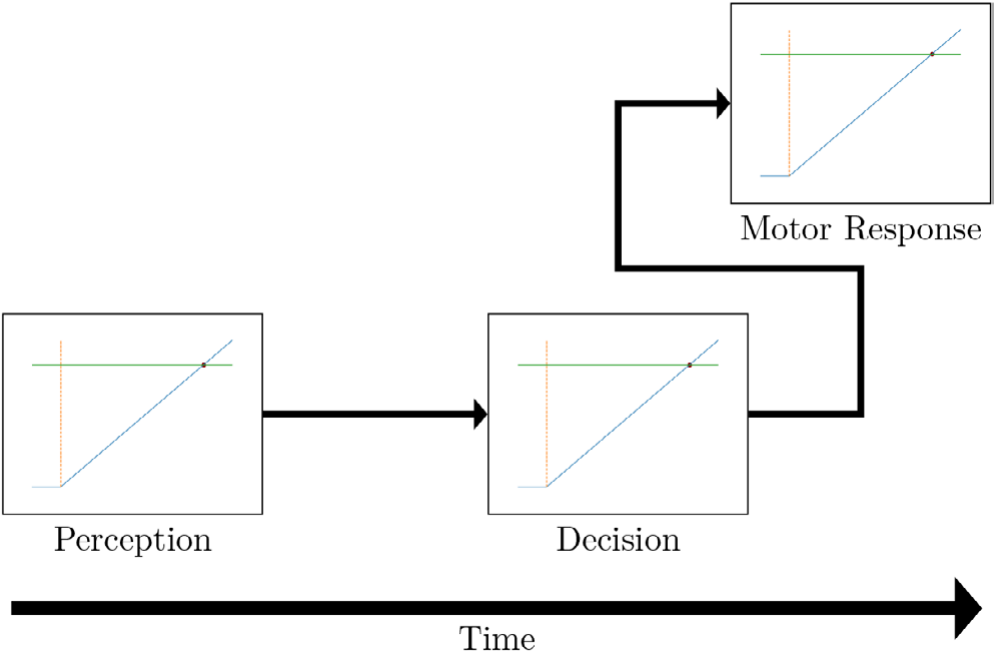
Illustration of the multi‐module LATER model, which includes a perceptual step, a decision step, and a motor response step. The decision step includes instruction processing, and the motor response may start before the decision step is complete.

Importantly, these works modeled SRT as a one‐step decision process, for instance by using a single LATER unit (i.e., the model described in Figure 1). Conversely, more recent studies have shown that the decision process can be better modeled through the use of multiple successive units, or modules; see Figure 2. (Carpenter et al., 2009; Reddi, 2001) have proposed to model the decision process itself as a two‐step model, with a perceptual module followed by a decision module. The former aims at the detection of whether a stimulus is present, as well as its main characteristic. As a consequence, this module has been found to be mostly influenced by the nature of the stimulus, and how easy it is to perceive, such as high contrast or luminance. The latter module then integrates the information of the first step (possibly from multiple stimuli) to interpret the information and decide which action is required. This second step has been shown to be influenced by more abstract elements, such as urgency or rewards (Carpenter et al., 2009). In particular, (Carpenter, 2004) has shown that expectation and unbalanced priors significantly influenced the decision module, and not the perceptual module. To reach this conclusion, they used an experimental design where the stimuli were clearly perceptible (high luminance/contrast). In this setting, the detection step was assumed to occur extremely fast, and thus the decision step became the predominant part of the reaction time and allowed the study of the impact of expectation on the second module.

In parallel, other works have also hypothesized that the decision step itself may include an instruction processing step (see e.g., (Sinha et al., 2006; Tari et al., 2022; Weiler & Heath, 2014) and references therein). In their experiments using switching tasks, the participants had to perform a (pro)‐saccade or an antisaccade, depending on the color of the fixation points, which was provided before the apparition of the stimulus. When the target color changed (switching condition), the reaction times were significantly longer, while when the subject was given enough time between the presentation of the task (the color hint) and the presentation of the stimulus, the SRT did not increase (Hunt & Klein, 2002). Together these observations point at the existence of an additional step detecting the instruction. Moreover, other works have also hinted at the existence of another step following the decision step, where motor responses are selected and planned. This step is often identified as the motor planning step (Horwitz & Newsome, 1999; Salinas et al., 2014), where the decision is converted into a motor response. Interestingly, following the elegant experimental framework of (Horwitz & Newsome, 1999), this step has been shown to sometimes start *before* the decision step is completed, in some specific settings. In other words, it is possible for the motor planning to begin before the target is chosen, after which the perceptual information, once detected, influences the motor plan (Diederich & Colonius, 2021).

In this paper, we investigate whether expectations may influence the instruction processing or the motor planning step. Indeed, while (Carpenter, 2004) attributed the impact of unbalanced priors to the decision step by ruling out the perceptual module, their model did not include either of the aforementioned additional steps. It is thus possible that expectations may impact one or more of these modules, and that the strength of the influence may depend on the complexity of the eye movement–both phenomena that could be missed when only studying saccades. For instance, if a more‐complex movement such as an anti‐saccade is highly anticipated, it is reasonable to expect that not only could the decision be faster, but moreover the motor planning step could also start even earlier, resulting in an even greater reduction of SRT. To study this possibility, we used a model for RT that contained both an instruction processing module, a decision module, and a motor response module (see Section 2.5 for more details), and we designed two experiments where both the expectation of location and the expectation of the type of movement (i.e., saccades or anti‐saccades) were manipulated to attempt to separate the impact of priors on each step of the decision process.

As aforementioned, and in line with previous studies on the influence of observer expectation (Anderson & Carpenter, 2006; Carpenter & Williams, 1995), we anticipated that the reaction times would decrease when both the target location and the type of eye movement become more probable. The goal of this experiment is to both quantify the changes in reaction times distribution as well as attribute these changes to different modules of the model LATER described previously.

## 2 MATERIALS AND METHODS

To assess our hypotheses, we conducted two different experiments. While both experiments shared the same experimental setup and the general characteristics of their stimuli, they all used different tasks (see below) and different groups of participants (to avoid any unwanted training/habituation effect (Wichmann & Hill, 2001)). The first experiment studied the influence of location priors on reaction times by manipulating the probability of the stimulus appearing on a given side, similarly to (Carpenter, 2004; Reddi et al., 2003). The study was performed for both saccadic movement (measuring Saccadic Reaction Times [SRT]) and antisaccadic movement (measuring Anti‐Saccadic Reaction Times [ART]). Importantly, in this experiment, there was no uncertainty regarding the type of movement to perform as saccades and anti‐saccades were grouped in separate blocks, and the observer was informed prior to each block of the type of movement to perform. The second experiment studied the influence of movement expectation on RT, using a mixture of both Saccades and Antisaccades. In this case, the observer did not know in advance which type of movement would be required for each trial. In particular we measured the SRT and ART of participants while manipulating the proportion of their respective type of movements.

All the experiments were approved by the Internal Review Board of the Department of Psychology of the University of Fribourg under the reference 2021‐749 R1, and were conducted in accordance with the declaration of Helsinki. Participants were recruited from the student population of the University of Fribourg through email‐list announcements, and each participant provided written informed consent prior to taking part in the experiments.

### 2.1 Experimental setup

The experiment was conducted in a dimly lit room on a 3.40GHz Intel(R) i7‐3770 CPU PC with Windows 10, on custom python 3.11 software built using the Psychopy library (Peirce, 2007). The stimuli were displayed on an EIZO FlexScan EV2451 screen (5 ms time latency) equipped with a Tobii Pro Spectrum eye tracker. Participants were seated 60 cm away in front of the screen, with the position of the head controlled using a chin rest fixed to the desk. Before any recording, the eye tracker was calibrated to the participant's vision using the Tobii software. The eye movements and the pupil diameter of the participants were recorded during the entire experiment.

### 2.2 Stimuli

In both experiments, the stimuli were high contrast high luminance in order to be clearly visible. All Stimuli were circles with radius of 1.5 degree of visual angle. The different stimuli possible were green (RGB 43:216:0) for saccades, red (RGB 255:0:0) for anti‐saccades or gray (RGB 225:225:225) for the fixation point. The background was dark gray (RGB 100:100:100) in order to maximize the visibility of the stimuli. Due to the high visibility of the stimuli, the detection step of the reaction process was considered negligible compared to the decision step (Reddi, 2001). Therefore, our analyses focused on the decision step, in which external information, such as prior probability of appearance, is assumed to be taken into account (Carpenter, 2004).

### 2.3 Experiment 1

Experiment 1 studied the influence of location priors on Saccadic Reaction Times (SRT) and Anti‐Saccadic Reaction Times (ART) by manipulating the probability of the stimulus appearing on a given side.

#### 2.3.1 Participants

24 healthy volunteers (19 males and 5 females) aged 20–35 (mean 24.9 y.o.) participated in the first experiment. They all had normal or corrected‐to‐normal vision. The general course of the experiment was explained to the participants, but they were naive to the objective of the analysis.

#### 2.3.2 Task

The experiment was divided into 10 blocks of 100 trials, separated by a 2 min pause. During each trial, participants were instructed to look at a gray circle located at the center of the screen until a fixation was confirmed by the eye tracker. Then, after a random waiting time between 400 and 1500 ms, another circle, either green or red, would appear either to the left or the right side of the fixation point, at a distance of 10 degrees. When the circle was green (resp. red), the participants were instructed to perform a saccade (resp. an antisaccade) toward (resp. in the opposite direction of) the new stimulus. The type of movement was known to the participant, as the 10 blocks were divided into two groups of five, one for the saccades and one for the antisaccades. The order of the two groups of blocks was randomly drawn at the beginning, and the results were communicated to the participant. The type of eye movement currently tested was also reminded to the participant at the beginning of each block. During each group of blocks, the proportion of stimuli appearing to the left and to the right varied, with respective values 100%–0% (only left), 80%–20%, 50%–50%, 20%–80%, and 0%–100% (only right) for each side, and half of the participants were going through these blocks in reverse order (only right first). The experiment was preceded by a training phase, where the participant could familiarize themselves with both tasks (saccades and antisaccades) in the equiprobable 50%–50% condition.

### 2.4 Experiment 2

Experiment 2 used a mixture of Saccades and Antisaccades and studied the influence of the proportion of each type of movement (saccade and antisaccade) on SRT and ART. Contrarily to Experiment 1, the probability distribution of the locations was kept uniform (50%–50%) throughout the experiment.

#### 2.4.1 Participants

24 different healthy volunteers (13 males and 11 females) aged 20–30 (mean 24.1 y.o.) participated in the second experiment. They all had normal or corrected‐to‐normal vision. The general course of the experiment was explained to the participants, but they were naive to the objective of the analysis.

#### 2.4.2 Task

The experiment was divided into five blocks of 200 trials, separated by a 2 min pause. Similarly to the previous experiments, for each trial, participants had to look at a gray circle located at the center of the screen until a fixation was confirmed by the eye tracker. Then, after a random waiting time between 400 and 1500 ms, another circle, either green or red, would appear either to the left or the right side of the fixation point, at a distance of 10 degrees. When the circle was green (resp. red), the participant was instructed to perform a saccade (resp. an antisaccade) toward (resp. in the opposite direction of) the new stimulus. Compared to the previous experiment, both colors were possible inside the same block, and the participant did not know in advance which type of eye movement would be required. The proportion of green and red stimuli depended on the block, with respective values 100%–0% (only saccade), 80%–20%, 50%–50%, 20%–80%, and 0%–100% (only antisaccade), with the order of the blocks being randomly drawn at the beginning. For each block both sides were equiprobable, meaning that 50% of the stimuli appeared on the left and 50% on the right. The experiment was preceded by a training phase, where the participant could familiarize themselves with the task, where stimuli included both saccades and antisaccades in the equiprobable 50%–50% condition.

### 2.5 Data analysis

All eye movements, as well as the size of the pupils, were recorded using the eye tracker. Pupil sizes were compared between blocks of the same experiment using a Friedman test for repeated measures to ensure that the arousal of participants did not vary significantly throughout the experiment.

For each experiment, block, type of movement (Saccadic and Antisaccadic), side (left and right), and reaction times (RTs) T were collected. First, RT measured outside the [60 ms, 800 ms] window were excluded from the analysis, in line with (Taylor et al., 2006). Second, $T_{\min}=60$ms were subtracted from each RT to account for incompressible transmission time (Noorani & Carpenter, 2015). Third, within each block we paid particular attention to the first 30 trials, called the early trials, as well as the trials after the 70‐th, called the late trials. Indeed, the early trials of a new block have been shown to reflect the expectation of the former block, while after 70 trials in the new condition, the observers have been deemed to have adapted to the new priors (Anderson & Carpenter, 2006). We thus compared the two distributions (early and late) using a Friedman test for repeated measures.

Each reaction time was then decomposed along the different steps of the perceptual decision process. Due to the high visibility of the stimuli, the detection step of the reaction process was considered negligible compared to the decision step (Reddi, 2001), and thus the corresponding module was not included in our model. Thus, the base model used in our analysis contained an instruction processing module, a decision module, and a motor response module, resulting in the following RT decomposition:

(1) $$T\approx T_{\min}+T_{\text{inst}}+T_{\mathrm{dec}}+T_{\text{motor}}$$

where $T_{\min}$ denotes the previously mentioned incompressible transmission time, $T_{\text{inst}}$ the time spent processing the instruction, $T_{\mathrm{dec}}$ the time used to decide a response, and $T_{\text{motor}}$ the additional time required to program a motor response. Importantly, depending on the experiment, not all steps were included in the analysis. In Experiment 1, the type of eye movement is known well in advance by the observer, thus canceling the need for an instruction processing (Hunt & Klein, 2002). Furthermore, when the location of the stimulus was known (late trials of the 100% one‐sided location block), the motor response could be fully planned in advanced, in which case we assumed that $T_{\mathrm{dec}}$ was the dominant term of the decomposition. Conversely, in Experiment 2, the location expectation was always uniform across all blocks (50% to the left, and 50% to the right). As a result, both the motor step and the decision step were assumed to be stationary across the different blocks for both saccade and anti‐saccade (i.e., the distribution does not depend on the block), and thus the variations of RT were assumed to mostly influenced by the instruction processing step. The different models are summarized in Table 1.

**TABLE 1:** Summary of the different decompositions of the variable reaction time ($T-T_{\min}$) used in the data analysis, depending on the experiment and block.

Task	Block	Decomposition ($T-T_{\min}$)	Notes
1	100%−0%	$\approx T_{\mathrm{dec}}$	‐
	0%−100%		
	Other	$\approx T_{\mathrm{dec}}$ + $T_{\text{motor}}$	
2	All	$\approx T_{\text{inst}}+T_{\mathrm{dec}}+T_{\text{motor}}$	$T_{\mathrm{dec}}$ and $T_{\text{motor}}$ are assummed stationary

#### 2.5.1 Bayesian models

In order to perform a Bayesian analysis, each resulting RT distribution was modeled using a Bayesian LATER model (Noorani & Carpenter, 2016), that is,

(2) $$\frac{1}{T-T_{\min}}\approx N\left(\mu_{B,M},\sigma_{B,M}\right),$$

where B,M denotes the dependency of the parameters with respect to the experimental setting, and respectively account for the **B**lock (e.g., movement and location expectation) and the type of eye **M**ovement (saccades versus antisaccades). We computed the posterior of all parameters $\mu_{B,M},\sigma_{B,M}$ using the respective non‐informative priors *U*([0.10]) (uniform distribution over the interval [0.10]) and *U*([0.20]) (uniform distribution over the interval [0.20]). Importantly, the LATER model is over‐defined with three parameters (Noorani & Carpenter, 2016), as any other set of parameters μ’, $\sigma^{\prime }$, and $\theta^{\prime }$ proportional to μ, σ, and θ would yield the same model. Hence, in our analysis we arbitrarily set θ=1, and as a result, experimental manipulation that would alter θ (e.g., divide it by 2) will impact simultaneously and equally impact μ and σ (e.g., multiply them by 2). Because expectation manipulation has been shown to impact θ in the LATER model, we mostly focused our analysis on the variations of the posteriors of μ, as μ is a useful proxy in that regard. The resulting posterior distributions were then used to provide the mean value of each parameter, together with the 95% High Density Interval (HDI). The distributions were also used to compare the parameters across the different blocks and condition, using the Bayes Factor which was estimated using the annealing sequential Monte Carlo sampling approach. Bayes Factor were interpreted using the scale detailed in Table 2. For the sake of convenience, when comparing populations, we also reported p‐values that were obtained using the non‐parametric Mann–Whitney *U*‐test with Bonferroni correction for multiple comparisons. All statistical analyses were performed using python 3.11, and the scipy and pymc libraries (Abril‐Pla et al., 2023).

**TABLE 2:** Summary of the interpretation of the Bayes Factor.

Bayes factor B	Interpretation
($H_{1}/H_{0}$)	
B≤0.001	Strong evidence in favor of $H_{0}$
0.001<B≤0.01	Moderate evidence in favor of $H_{0}$
0.01<B≤0.1	Some evidence in favor of $H_{0}$
0.1<B<10	No evidence
10≤B<100	Some evidence in favor of $H_{1}$
100≤B<1000	Moderate evidence in favor of $H_{1}$
1000≤B	Strong evidence in favor of $H_{1}$

## 3 RESULTS

### 3.1 Experiment 1: Preliminary analysis

The pupil diameter of the observers were not found to vary significantly between the different conditions (p=0.999). As pupil size is considered a gold standard to measure arousal in subjects in a controlled environment, this result indicates that the attention of the participants did not change significantly during the experiment. Similarly, when comparing left and right side stimuli across matching priors (e.g., left stimuli in the 80% left vs. 20% right block, compared to right stimuli in the 80% right vs. 20% left block), no statistically significative difference between RTs was found (p=0.216 for saccades, p=0.356 for antisaccades). Consequently, RTs from both sides were aggregated for the next steps of the analysis.

Interestingly, when comparing the early trials of each block (e.g., first 30 trials) to the late trials of the same block (e.g., after 70 trials), no statistically significant difference between RTs were found in most cases. More specifically, only the 100%–0% distribution (one‐sided block) yield a difference (p=0.027 for antisaccades and p=0.006 for saccades). This result differed from the observation of (Anderson & Carpenter, 2006). Nevertheless, we selected the late trials of each block for the rest of the analysis, in line with the aforementioned study.

**FIGURE 3:**
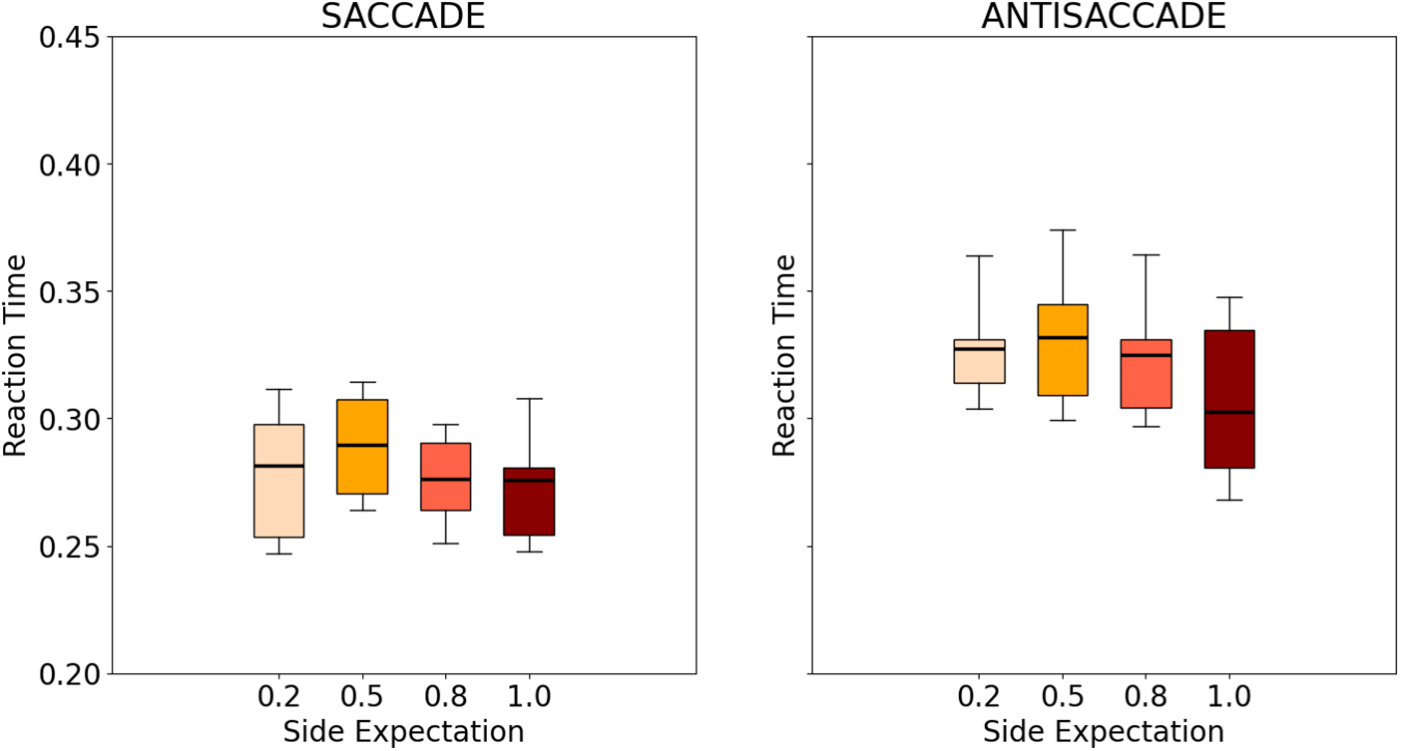
Distribution of the RT of participants for each combination of stimuli side distribution and type of eye‐movement in Experiment 1. The line, block, and whiskers represent respectively the median, 25%–75% percentile, and 5%–95% percentile of the distribution. Only the late trials of each block were considered.

Figure 3 shows the distribution of the average reaction times (RT), for each block (100%–0%, 80%–20%, 50%–50%, and 100%–0% distributions) for both saccades and antisaccades. As expected, ART was larger than SRT, which can be explained by the fact that antisaccades are more complex responses compared to saccades which are highly optimized and automated, see for example, (Kveraga et al., 2002). Overall, no pattern appeared in this preliminary analysis: RT did not appear to be strongly influenced by the side expectation. A small difference may be noticed for the 100%–0% block, which corresponds to the case where the stimulus always appears in the same place (and thus, both the type and the direction of the expected eye movement are always the same).

### 3.2 Experiment 1: Bayesian analysis

**FIGURE 4:**
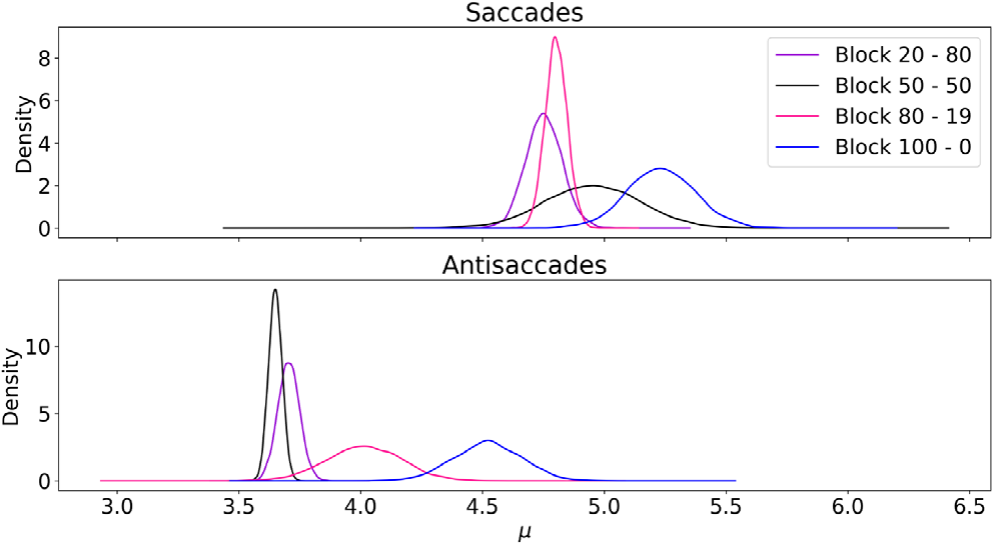
Posterior distribution of the parameter μ (average information rate) for each combination of stimuli side distribution and type of eye movement in Experiment 1. Only the late trials of each block were considered.

Figure 4 shows the posterior distribution of μ, that is, the average rate of information acquisition, for each block (100%–0%, 80%–20%, 50%–50%, and 100%–0% distributions) for both saccades and antisaccades. Overall, a small pattern emerged in many participants: the more likely a specific side was, the higher the corresponding μ (and thus, the faster the corresponding average RT tended to be). This phenomenon was more clearly visible for the 100%–0% block, and the differences between the other blocks were smaller. Table 3 reports the characteristics of the posterior distribution for the difference between the RT of different blocks, for each type of eye movement, as well as the corresponding Bayes Factor. Interestingly, the quantitative analysis reproduced the observation of the descriptive analysis. First, and perhaps surprisingly, no evidence of difference was found between the 20%–80%, 50%–50%, and 80%–20% blocks for the saccadic movement (BF 0.74 and 0.23). While this observation appears to be in contradiction with previous results, these results may stem from the fact that this experiments used more subjects than previous studies, while having less trials per subjects. This phenomenon is also reflected in both the average value of the difference which was small (−0.05 and −0.142 s^−1^), while both 95% high density interval –HDI– included both negative and positive value ([−0.623, 0.578] and [−0.478, 0.292]). These results point to the fact that different behavior could be observed in our data, and that some observers reduced their RT while others increased them between blocks. Second, some evidence was found of a difference between the 100%–0% block and the 80%–20% block (BF 17.7). Importantly, the difference was larger than the previous one but still small (0.35 s^−1^, 95% HDI [0.02, 0.751]), pointing at a small improvement of the reaction time. The analysis of the antisaccades exhibited a different pattern. First, some evidence was found of a difference between the 20%–80%, 50%–50%, and 80%–20% blocks (BF 20.1 and 0.02). The differences appeared larger regarding their average value (0.544 and −0.186 s^−1^), however their HDI still included both negative and positive value ([−0.436, 0.129] and [−0.308, 0.055]), which limits the strength of these findings. Second, there was moderate evidence that ART were smaller in the 100%–0% block than in the 80%–20% block (BF 105.0). The difference of rate of information acquisition was also larger, with average value 0.63 s^−1^ and HDI [0.010, 1.193] Overall, in both saccades and antisaccades conditions, the difference between the 100%–0% block and the 80%–20% block was larger than the other differences. This result could be explained by the fact that in the 100%–0% condition, while the delay before the appearance of the stimulus is random, the motor response is always the same (as the stimuli always appear on the same side, and the required type eye movement is known before the trial), and thus can be planned in advanced.

**TABLE 3:** Characteristics of the posterior distribution of μ for the difference between the different blocks, for each type of eye movement in Experiment 1.

Movement	Comparison	Avg value	95%‐HDI	BF	*p*‐value
Saccades	$\mu_{100-0}-\mu_{80-20}$	0.35	[0.002, 0.751]	17.7*	0.039
	$\mu_{80-20}-\mu_{50-50}$	−0.05	[−0.623, 0.578]	0.74	0.807
	$\mu_{50-50}-\mu_{20-80}$	−0.142	[−0.478, 0.292]	0.23	0.058
Anti‐Saccades	$\mu_{100-0}-\mu_{80-20}$	0.63	[0.010, 1.193]	105.0**	<0.001
	$\mu_{80-20}-\mu_{50-50}$	0.544	[−0.436, 0.129]	20.1*	0.001
	$\mu_{50-50}-\mu_{20-80}$	−0.186	[−0.308, 0.055]	0.02*	0.014

*Note*: Avg Value reports the average value of the posterior distribution, HDI the 95% high density interval, BF the Bayes Factor of the hypotheses “The rate of acquisition increased” versus “The rate of acquisition decreased”. No stars (resp. *, **, ***) indicates no evidence (respectively some evidence, moderate evidence, strong evidence). The table also reports the *p*‐value resulting from a non‐parametric Mann–Whitney *U*‐test for convenience.

### 3.3 Experiment 2: Preliminary analysis

Similarly to Experiment 1, no significant differences in pupil diameter were observed between the various experimental conditions (p=0.821), suggesting that participants' attention levels remained stable throughout the experiment. Moreover, a comparison of response times (RT) between stimuli presented on the left and right sides for the same eye movement also revealed no statistically significant differences across corresponding distribution blocks (e.g., left saccades versus right saccades in an 80% saccade vs. 20% antisaccade block) (p=0.706). Consequently, RTs from both sides were pooled for subsequent analyses. When examining early versus late trials within each block (e.g., comparing the first 30 trials to those occurring after 70 trials), no statistically significant differences in RT were found in any block (p=0.180). Nevertheless, to ensure consistency, the late trials of each block were selected for further analysis.

**FIGURE 5:**
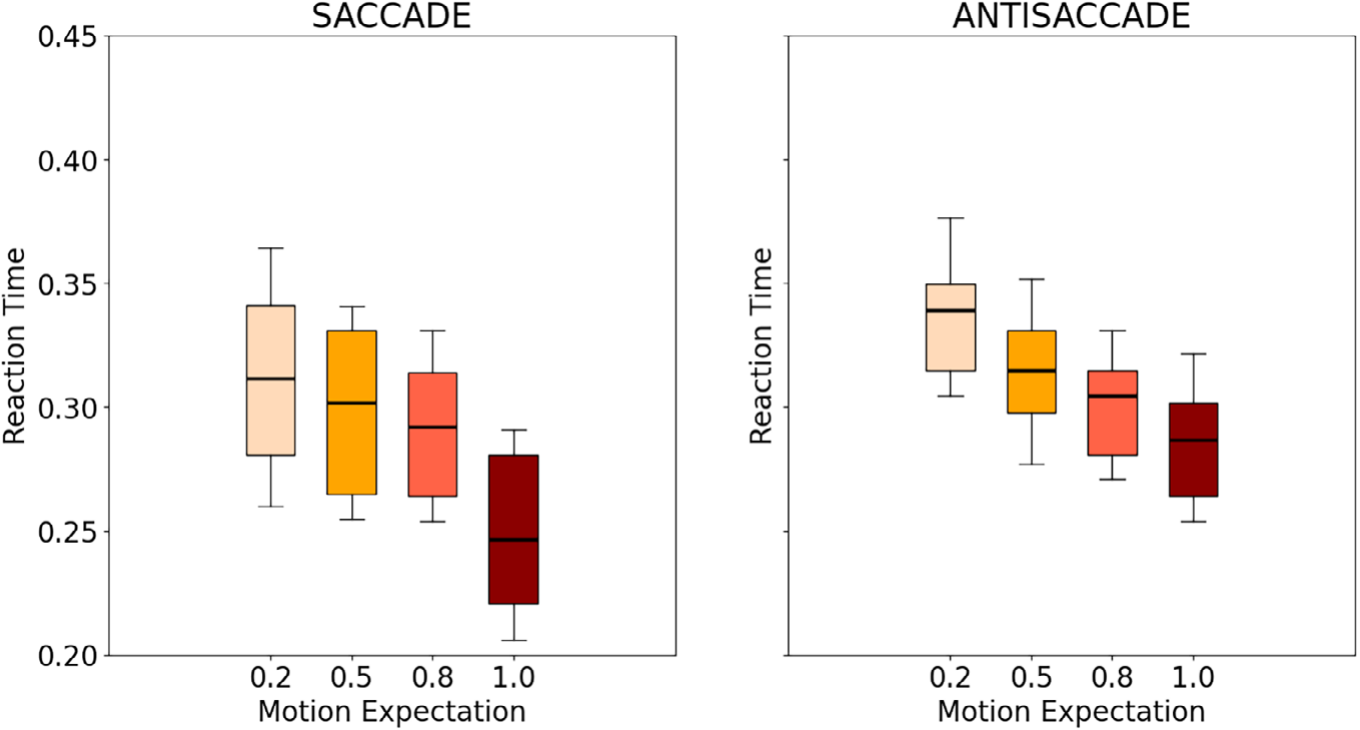
Distribution of the RT of participants for each combination of expectations of the type of movement (motion expectation for short) in Experiment 2. The line, block, and whiskers represent respectively the median, 25%–75% percentile, and 5%–95% percentile of the distribution. Only the late trials of each block were considered.

Figure 5 shows the distribution of RT for each block (100%–0%, 80%–20%, 50%–50%, and 100%–0% distributions) for both saccades and antisaccades. As anticipated, ART were also longer than SRT, in line with the previous results as noted in (Kveraga et al., 2002). Importantly, a more pronounced pattern emerged across all participants, compared to Experiment 1: the higher the probability of a particular type of eye movement, the higher the corresponding value of μ. This difference between blocks also appears larger for both saccades and antisaccades, with the variances of the posterior distribution smaller in this setting.

### 3.4 Experiment 2: Bayesian analysis

**FIGURE 6:**
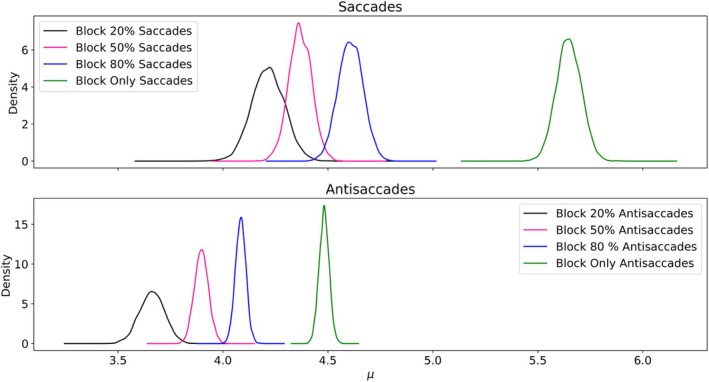
Posterior distribution μ (average information rate) for different eye movement expectations in Experiment 2. Only the late trials of each block were considered.

Figure 6 presents the posterior distribution of μ, representing the average rate of information acquisition, for each block (100%–0%, 80%–20%, 50%–50%, and 100%–0% distributions) for both saccades and antisaccades. Importantly, this figure also highlights the stronger pattern noted in Figure 5. This difference between blocks appears larger for both saccades and antisaccades, with the variances of the posterior distribution smaller in this setting. Table 4 summarizes the characteristics of the posterior distribution for the differences between reaction times (RT) across various blocks for each type of eye movement, as well as the corresponding Bayes Factor (BF). Firstly, and compared to Experiment 1, there is strong evidence of a difference in RT between all the blocks, for both eye movements (with the smallest Bayes Factor being $10^{6}$). Additionally, for saccades the impact of the movement prior appeared larger than the impact of location prior studied in Experiment 1: the average difference between the 100%–0% block and the 80%–20% block was 1.068 compared to 0.35, between the 80%–20% block and the 50%–50% block 0.324 compared to −0.05, and between the 50%–50% block and the 20%–80% block 0.562 compared to −0.142. The same effect was also observed in the HDI, which were significantly narrower in Experiment 2. Importantly, the expectation of location was always uniform in Experiment 2, and as a result, both positions were equally likely to be the target of the eye movement, regardless of the type of eye movement (saccade or antisaccade) or their respective expectation. As a result, both motor responses (looking to the left or looking to the right) were equally likely throughout the experiment, and the differences of RT are likely to be caused by gains in the instruction processing module. This remark is buoyed by the quantitative analysis of the ART. Indeed, while there were also strong evidence for the differences between the blocks, the amplitude of these differences were more similar to the one observed in Experiment 1: the average difference between the 100%–0% block and the 80%–20% block was 0.509 compared to 0.63, between the 80%–20% block and the 50%–50% block 0.229 compared to 0.544, and between the 50%–50% block and the 20%–80% block 0.611 compared to 0.186. Moreover, the HDI were also narrower than in Experiment 1, pointing at a more uniform behavior across the observers. Finally, the differences between blocks were similar for both saccades and antisaccades (except for the expectation 100%–0% for saccades). These results hint at the existence of a common mechanism behind these differences, which may be explained by the impact of the instruction processing step.

**TABLE 4:** Characteristics of the posterior distribution of μ for the difference between the different blocks, for each type of eye movement in Experiment 2.

Movement	Comparison	Avg value	HDI	BF	*p*‐value
Saccades	$\mu_{100-0}-\mu_{80-20}$	1.068	[0.898, 1.241]	$10^{30}$***	<0.001
	$\mu_{80-20}-\mu_{50-50}$	0.324	[0.182, 0.455]	$10^{6}$***	<0.001
	$\mu_{50-50}-\mu_{20-80}$	0.562	[0.343, 0.791]	$10^{6}$***	<0.001
Anti‐Saccades	$\mu_{100-0}-\mu_{80-20}$	0.509	[0.433, 0.584]	$10^{34}$***	<0.001
	$\mu_{80-20}-\mu_{50-50}$	0.229	[0.137, 0.315]	$10^{7}$***	<0.001
	$\mu_{50-50}-\mu_{20-80}$	0.611	[0.475, 0.754]	$10^{14}$***	<0.001

*Note*: Avg Value reports the average value of the posterior distribution, HDI the 95% high density interval, BF the Bayes Factor of the hypotheses “The rate of acquisition increased” versus “The rate of acquisition decreased”. No stars (resp. *, **, ***) indicates no evidence (respectively some evidence, moderate evidence, strong evidence). The table also reports the *p*‐value resulting from a non‐parametric Mann–Whitney *U*‐test for convenience.

## 4 DISCUSSION

### 4.1 Expectation of location have a limited impact on SRT

First, it is interesting to note that the results on SRT observed in XP1 differed from previous studies on the same topic. More precisely, no evidence of differences were found between the rate of information acquisition of blocks corresponding to the 80%–20%, 50%–50%, and 20%–80% conditions (Bayes Factor between 0.1 and 10). Moreover, there was only limited evidence of a difference between the 100%–0% and the 80%–0% blocks (Bayes factor 17.7), for an average of the μ posterior of respectively 5.25 and 4.9, corresponding to roughly 15 ms of difference between the SRT. This is in stark contrast with for example, (Carpenter & Williams, 1995), which noted a significant −80 ms of SRT per log unit of expectation (for reference, the difference between the 50%–50% and 20%–80% conditions is approximately 1 log unit). This discrepancy may stem from different factor. First, in our experiments, each participant only performed 1000 trials, which is dwarfed by (Carpenter & Williams, 1995) and (Anderson & Carpenter, 2006), where the main observers made more than 100,000 saccades. It is possible that the SRT are particularly difficult to model due to a small signal‐to‐noise ratio, and thus the difference between the different block in our experiment is masked by high noise levels. However, such explanation would conflict with the strong effect of expectation manipulation measured by (Carpenter & Williams, 1995) (−80 ms per log unit). It is also possible that the habituation process is long, that is to say each observer requires numerous trials in the same condition before updating their own expectation. Indeed, in (Carpenter & Williams, 1995), the change in the profile of saccadic latencies took “many hour,” according to the authors. While possible, this result would be at odds with (Anderson & Carpenter, 2006), where the authors observed that 70 trials was enough for the distribution of SRT to be fit to the new location expectation. Second, our experiments involved a larger number of subject (more than 20), compared to the two observers of (Carpenter & Williams, 1995) and (Anderson & Carpenter, 2006). Consequently, it is also possible that the observations of the aforementioned works may not be true for every individual. Indeed, the large variance of the posterior of μ in our study hint at a range of responses from observers when manipulating expectations in Experiment 1. Finally, it is important to note that this result is in line with the observations of (Basso & Wurtz, 1998), who measured a ≈20 ms difference in the ape reaction time between the uniform uncertainty (corresponding to the 50%–50% block in Experiment 1) and the no uncertainty conditions (corresponding to the 100%–0% block in Experiment 1). It is however important to note that their study used animal observers, in opposition to our experiment.

### 4.2 Expectation of location have a larger impact on ART

Interestingly, ART exhibited a more pronounced pattern in our experiments. Indeed, there were limited evidence of a relation between location expectation and the rate of information acquisition among blocks corresponding to the 80%–20%, 50%–50%, and 20%–80% conditions (Bayes Factor 20.1 and 0.02, respectively). There was also moderate evidence of a difference between the 100–0 and the 80–0 blocks (Bayes factor 105), for an average of the μ posterior of respectively 4.6 and 4.0, corresponding to roughly 40 ms of difference between the ART. Interestingly, these values are more in line with the value noted by (Anderson & Carpenter, 2006) for SRT. Importantly, the decision process was similar between the saccade and the anti‐saccade conditions (similar expectations), and only the motor response differed. As the gains from expectation appear larger in the anti‐saccade condition, these gain may hint at different optimization of the motor response. Indeed, as noted by previous studies (Salinas et al., 2014), the programming of the motor response can start before the perceptual decision is made. Thus, when a particular location is more likely (and therefore, a particular motor response), the programming can start early resulting in a likely faster RT. The fact that the difference was larger in ART compared to SRT may be explained by the fact that saccades are highly optimized movement, that naturally occur several times per second in humans (Ibbotson & Krekelberg, 2011). Conversely, anti‐saccades are not an ecological eye movement, which takes significantly longer to perform among observers which are not used to them (Kveraga et al., 2002). Therefore, planning an anti‐saccade in advance due to high likelihood of a stimulus appearing at a given location (that is to say, unbalanced expectations) may reduce this latency.

### 4.3 Expectation of type of movement have a strong impact on both SRT and ART

In experiment 2, where the expectation of the type of eye movement was manipulated (instead of expectation of location), we observed strong differences between the different blocks. There was strong evidence for a relationship between expectation and the rate of information acquisition among all the blocks, and for both types of eye movements (BF $>10^{6}$). Futhermore, the differences were larger, with for instance an average of the μ posterior of respectively 5.7 and 4.6 between the 100%–0% and the 80%–20% blocks for saccadic movements, corresponding to roughly 42 ms of difference between the SRT. These results are in line with previous observations regarding the cost of task‐switching (see e.g., (Sinha et al., 2006; Tari et al., 2022; Weiler & Heath, 2014)): when the type of movement is predictable (which corresponds to the 100%–0% block in our experiment), observers achieve significantly smaller RT. Moreover, our results also point to a dose‐effect phenomenon, where the more unpredictable the type of movement (e.g., the higher the entropy of the distribution) the longer the RT, which, to the best of our knowledge, has not been studied before in this setting. This was observed for both saccades and anti‐saccades. Importantly, the expectation of location (left–right) was constant throughout this experiment, that is, the target of the eye movement was equally likely to be on either side. As a consequence, it is unlikely that these differences may be explained by the motor response module, as both possible trajectories were always equally propable. Thus, the most likely source of this difference is the instruction processing unit. Altogether, these findings hint at the fact that both motor response and instruction processing units were influenced by expectations. As a result, we argue that previous works that attributed the entire effect to the decision process may have missed more subtle effects, due to the interaction of the impact on the different modules.

### 4.4 Alternative models of RT

The observed latency differences in Experiment 2 may partly reflect the need to override a dominant expectation when the minority movement type is required (block 80%–20%). As a result, these observations could be modeled with a stop or inhibition process, as for example, in (Hanes & Carpenter, 1999). However, we argue that such models would be less useful for Experiment 1 and the other blocks in Experiment 2, where either all the movements are of the same type or both types of movements are equally probable. Since this study aimed at analyzing the differences between RT across different blocks, this work used a model that was deemed to be equally well‐suited to each experiment. Nevertheless, we acknowledge that this explanation could provide a complementary approach to our account based on instruction‐processing duration, and future work could incorporate an explicit inhibitory mechanism into the model to help distinguish between increased instruction processing time and the cost of suppressing a prepotent response.

### 4.5 Limitations

Despite the careful design of our experiments, several limitations must be acknowledged. First, the relatively limited number of trials per participant (1000 trials) may have affected the statistical power of our analyses, especially when compared to some previous studies where observers performed over 100,000 saccades. This limitation, which was necessary in order to measure more than 20 observers, may have contributed to the high variability observed between individuals and may obscure subtle effects of expectation on reaction times, in particular for Experiment 1. Indeed, it is possible that the limited number of samples reduced the precision of the Bayes Factor estimate, resulting in an incorrect lack of evidence. Additionally, the relatively short duration of each block (100–200 trials) may not have been sufficient for participants to fully acclimate to the new expectations. Indeed, past studies reached different conclusions in that regard, and some (see e.g., (Carpenter & Williams, 1995)) suggested that prolonged exposure is necessary for stable adaptation to occur for saccadic movements. Furthermore, this small number of trials limited the possibility to accurately estimate the parameters of multiple cascaded accumulators from RT. However, it should be noted that we designed the experiments to isolate a specific unit, similar to (Carpenter et al., 2009), which alleviated this problem. Importantly, and despite these possible limitations, the effect of movement expectation manipulation was very significant in our experiments (Experiment 2). Finally, we focused on saccadic and anti‐saccadic eye movements, and thus our findings may not necessarily extend to other types of movements or decision‐making contexts.

## AUTHOR CONTRIBUTIONS

Julien Audiffren conceived and designed this research study, performed experiments, analyzed data, and interpreted results of experiments. Julien Audiffren prepared figures and drafted the manuscript. Julien Audiffren, Jean‐Luc Bloechle, and Jean‐Pierre Bresciani edited, revised, and approved the final version of the manuscript.

## FUNDING INFORMATION

This study was supported by funding provided by the University of Fribourg.

## CONFLICT OF INTEREST STATEMENT

The authors declare no conflicts of interest.

## ETHICS STATEMENT

All participants provided written informed consent. This study was conducted in accordance with the Declaration of Helsinki and received approval from the Internal Review Board of the Department of Psychology of the University of Fribourg under the reference 2021‐749 R1.

## Data Availability

The data obtained and analyzed in this study can be obtained upon request to the corresponding author.
